# Implementation of task‐based fMRI in TRAILBLAZER‐ALZ 6 Phase 3b study: Methodological considerations

**DOI:** 10.1002/alz70856_105633

**Published:** 2026-01-09

**Authors:** Chris Conklin, Stefan Radonjic, David Scott, Madhura Ingalhalikar, Luc Bracoud, Diana O Svaldi, Hong Wang, Emel Serap Monkul Nery, Sergey Shcherbinin, John R. Sims, Mark A. Mintun, Emily C. Collins

**Affiliations:** ^1^ Clario, Inc., Philadelphia, PA, USA; ^2^ Clario, Inc., San Mateo, CA, USA; ^3^ Clario, Inc., Lyon, France; ^4^ Eli Lilly and Company, Indianapolis, IN, USA

## Abstract

**Background:**

Task‐based functional Magnetic Resonance Imaging (tb‐fMRI) leverages blood oxygenation to measure the brain's response to external stimuli. A lack of tb‐fMRI equipment standardization is the well‐recognized challenge when deploying this promising approach in multi‐center clinical trials. We sought to implement and characterize visual task‐based brain activation as an exploratory measurement in TRAILBLAZER‐ALZ 6 (NCT05738486), a multicenter, randomized, double‐blind, Phase 3b study of donanemab in adults with early symptomatic AD and confirmed amyloid pathology.

**Method:**

A total of 41 3 Tesla scanners were qualified for tb‐fMRI, including 9 sites with established hardware (e.g. MR compatible Liquid Crystal Displays / goggles and triggering devices) and 32 sites lacking hardware *(de novo)* where a video‐capable projector and an MRI coil mirror were provided. A 4‐minute visual task (alternating 30s blocks of flashing annulus and crosshair) was disseminated either via a paradigm *(established)* or as a video *(de novo)*. fMRI acquisition was an interleaved gradient recalled echo planar (GRE‐EPI) sequence with TR(repetition time)/TE(echo time)= 3000ms/30ms, voxel size = 3x3x4mm^3^, and 35 slices. fMRI data was pre‐processed and spatially normalized to brain atlas space using a validated Clario pipeline. Processed fMRI data was input to a general linear model (GLM) with outlier regression. Brain activation for each scan was quantified as the mean T‐score and Blood Oxygenation Level Dependent (BOLD) contrast within a pre‐specified region of interest (ROI) of atlas‐space occipital subregions.

**Result:**

Task‐based fMRI data was acquired from 799 participants (*n* *established* = 192, n *de novo* = 607). Brain activation within the entire ROI was significant across all participants for mean T‐score (*t* > 7, *p* < 0.0001) and BOLD contrast value (*t* > 6.4, *p* < 0.0001). At the group‐level, participants scanned on *established* equipment had significantly higher BOLD contrast values relative to *de novo* sites (two‐tailed *p* < 0.03), but mean T‐scores were not significantly different.

**Conclusion:**

Task‐based fMRI is viable in multi‐center clinical trials. Novice sites can be trained to utilize off‐the‐shelf components and generate analyzable data. Variance due to fMRI equipment capabilities appears to impact the magnitude of ROI activation more than its significance level.

